# Cross-National Gender Gaps in Political Knowledge: How Much Is Due to Context?

**DOI:** 10.1177/1065912916642867

**Published:** 2016-04-13

**Authors:** Jessica Fortin-Rittberger

**Affiliations:** 1 University of Salzburg, Austria

**Keywords:** political knowledge, gender, electoral institutions, women and politics, survey research

## Abstract

Although the majority of studies on political knowledge document lingering gender-based differences in advanced industrial democracies, most contributors have drawn such conclusions from a single or a handful of countries, using limited batteries of political information items. Exploiting a pooled data set of the Comparative Study of Electoral Systems encompassing 106 post-election surveys in forty-seven countries between 1996 and 2011, this article demonstrates that survey instrument–related factors, such as question format and content, as well as the overall difficulty of questions, are more consequential in shaping the size of gender gaps in political knowledge than institutional factors, such as electoral rules or opportunity structures. The research design of this article draws from almost three hundred different items measuring factual political knowledge using the broadest country coverage and most comprehensive approach to measurement to date.

## Introduction

Political knowledge is a central indicator of “cognitive engagement” underpinning attitude formation and connectedness to political processes (Zaller 1992). In short, political knowledge is a crucial resource for citizens to participate effectively in politics (Basinger and Lavine 2005; Gelman and King 1993; Lau and Redlawsk 2006; Sniderman, Glaser, and Griffin 1991). Cognitive engagement is considered to be essential for democratic citizenship, yet political knowledge is unequally distributed: the overwhelming majority of studies on the topic finds a sizable and consistent gap between men and women on batteries of items measuring political knowledge (Burns, Schlozman, and Verba 2001; Delli Carpini and Keeter 1996, 2000; Dow 2009; Fraile 2014; Frazer and Macdonald 2003; Garand, Guynan, and Fournet 2005; Kenski and Jamieson 2000; Lizotte and Sidman 2009; Mondak and Anderson 2004; Sanbonmatsu 2003; Verba, Burns, and Schlozman 1997; Wolak and McDevitt 2011).

Although the existence of gender-based differences in political knowledge is an established finding, most conclusions about their causes were reached from studies focusing on only a single or a handful of countries and have mainly highlighted individual-level predictors.^1^ To date, no comparative study has sought to combine the different individual and contextual—societal, institutional, and instrument-related—variables that significantly affect the difference in political knowledge between men and women across several countries. This article seeks to address this lacuna by investigating whether contextual cross-national differences hold a key to explain these gender gaps. Drawing on a pooled data set containing three modules of the Comparative Study of Electoral Systems (CSES) data project, this analysis uses the broadest country coverage and most comprehensive approach to measurement to date.

This article sets out to make two contributions: first, using three different scoring systems to deal with factual knowledge questions—measuring positive knowledge, expressive answers, and levels of accuracy—the analyses provide evidence of pervasive gender gaps in political knowledge that hold for more than forty countries irrespective of the approach to measurement employed. The second contribution stems from the differentiated approach to measurement. The magnitude of gender gaps is contingent on scoring systems used by researchers: considering “don’t know” (DK) responses as incorrect systematically penalizes women. Moreover, survey instrument–related factors, such as question format and content, as well as survey mode, influence the number of correct responses and the level of accuracy of female respondents. The survey mode also heavily impacts the likelihood of women giving an expressive answer, no matter if correct or incorrect. By contrast, institutional variables, for example, electoral rules or opportunity structures for women, do not seem to play a significant role in explaining the different performance of men and women. The analyses therefore suggest that more causally proximate variables, such as survey instruments, exert a more direct influence on respondents than broader contexts.

## Gendered Differences in Political Knowledge

The overwhelming majority of studies exploring the differences in political knowledge across genders document a sizable and consistent gap between the performance of men and women on batteries of items measuring political knowledge, indicating that women are less knowledgeable about politics than men (Burns, Schlozman, and Verba 2001; Delli Carpini and Keeter 1996, 2000; Dow 2009; Fraile 2014; Frazer and Macdonald 2003; Garand, Guynan, and Fournet 2005; Kenski and Jamieson 2000; Kittilson and Schwindt-Bayer 2012; Lizotte and Sidman 2009; Mondak and Anderson 2004; Sanbonmatsu 2003; Verba, Burns, and Schlozman 1997; Wolak and McDevitt 2011). To account for these persistent disparities, existing research has advanced three types of hypotheses: the first set of explanations focuses on the survey questions used to measure political knowledge, more precisely, the content and format of these questions. The second type of explanation focuses on the role of women in society and the resources at their disposal, and the third group of explanations addresses the influence of political institutions, such as electoral systems.

The first set of contributions, focusing on the form of the survey instruments employed, posits that the type of questions measuring political knowledge asked in surveys is part of the explanation for the differences in political knowledge between men and women.^2^ One potentially important difference is located at the individual level. Men and women are considered to exhibit different psychological tendencies when answering questions: men are hypothesized to display a higher “propensity to guess” than women, who in turn are more likely to select DK when they are insecure (Atkeson and Rapoport 2003; Frazer and Macdonald 2003; Kenski and Jamieson 2000; Lizotte and Sidman 2009; Mondak and Anderson 2004; Mondak and Canache 2004). This gender-based difference in the propensity to guess affects survey results: closed-ended questions are understood to elicit more “guessing” that open-ended questions. Thus, results from different question formats are subject to different biases that affect the gender gap size (Luskin and Bullock 2011; Sturgis, Allum, and Smith 2008).

A second set of contributions focuses on the societal structures that shape the acquisition of knowledge. One branch of research posits that part of the explanation for the gender gap lies in the types of questions contained in surveys. Contingent on their life experience, men and women acquire different knowledge (Dolan 2011; Norris 2000; Shaker 2012; Smiley 1999; Stolle and Gidengil 2010; Verba, Burns, and Schlozman 1997), so tapping only a particular type of knowledge results in considerable gender bias. The predominant use of knowledge questions in present-day surveys, asking, for instance, for information about politicians in specific positions (e.g., the name of specific ministers), political parties (i.e., their ideological position and coalition partners), or technical rules concerning political processes (such as term limits or the number of parliamentarians), gives undue privilege to “male” knowledge. Women’s practical knowledge of government, which is more focused on public services and welfare state policies, is rarely evoked in surveys. In other words, survey item selection might put women at a structural disadvantage (Dolan 2011). The underlying assumption of this branch of research is that different patterns of socialization shape the levels of attention men and women devote to politics. The standard explanations center on differential socioeconomic and cognitive resources available to men and women. The usual suspects are political interest, media attentiveness, education, socialization, and other equivalent indicators underlying political motivation (Banwart 2007; Bennett and Bennett 1989; Delli Carpini and Keeter 1996; Dow 2009; Flora and Lynn 1974; Frazer and Macdonald 2003; Gidengil and Thomas 2008; Jennings 1983; Price and Zaller 1993; Wolak and McDevitt 2011).

A third and more recent set of analyses has zoomed in on political institutions as potential mediating influences on women’s level of political knowledge. Kittilson and Schwindt-Bayer (2012) hypothesized that more proportional electoral rules provide additional incentives for political parties to mobilize women and that this mobilization affects their level of engagement, which in turn influences their overall knowledgeability.

Taken together, these studies provide important clues about the covariates of the gender gap in political knowledge. However, given the scarcity of comparative studies on gendered differences in political knowledge, we have had little opportunity to test these explanations concurrently. The present article covers some of these blind spots by testing these explanations of differences in political knowledge using the broadest country coverage and most comprehensive approach to measurement to date.

## Data and Method

The data for this article are drawn from the countries included in the three modules of the CSES project and cover the period 1996–2011 (CSES 2003, 2007, 2013). Although CSES data are mainly centered on advanced industrial democracies, they also include a number of newer democracies in which competitive elections are held, and thus display a broad geographic coverage including countries in Asia and Latin America. The pooled modules include 106 post-election surveys conducted in forty-seven countries (see the online appendix, Table I, for details at http://prq.sagepub.com/supplemental/). Given the strategic timing of CSES interviews, fielded on average within three months after an election has taken place, this study provides an optimal analytical context in which the most recent elections are roughly equally salient to respondents. The individual-level data from surveys were supplemented by aggregate-level data drawn from a variety of sources, detailed below.

The analyses proceed in two steps. First, macro-level analyses estimating the size of the gender gap in political knowledge (aggregated for each election study) are presented. In a second step, a series of multilevel ordered logistic regressions is used to estimate the effects of both individual-level and macro-level variables on individuals’ levels of political knowledge. This research design thus harnesses the strength of both macro-level and multilevel cross-national empirical verifications to account for gendered differences in political knowledge.

### Measuring Political Knowledge

The CSES contains three questions per election study measuring political knowledge, each displaying different degrees of difficulty, mainly tapping into the traditional understanding of political information (such as identifying political officeholders or officials) and verifying the extent of knowledge on key institutions (such as the size of assemblies or details concerning electoral rules). These items are not standardized across countries, meaning that each national election study team is left free to ask these questions according to their national standards. Although the lack of standardization makes direct comparison across countries challenging, most items are similar enough in content to permit some valuable assessments. Each set of questions contains a correct answer, which is clearly identified in the codebook.

Following the recommendation by Frazer and Macdonald (2003), this contribution uses three different additive indices of political information. Exploiting three scoring systems allows tackling the presence of random components in answer patterns head-on by addressing the issues of “response bias” around the DK category, as these answers are of particular importance when comparing men and women (Kenski and Jamieson 2000; Mondak and Anderson 2003; Verba, Burns, and Schlozman 1997).^3^

The first operationalization, coined *Positive Knowledge Scale*, only codes correct answers to a question as 1; the remainder categories, both incorrect and DK answers, are coded 0. This way of coding answers only captures positive political knowledge and therefore assumes that knowledge is discrete, with respondents holding this attribute or not, which some consider to be problematic (Delli Carpini and Keeter 1996; Mondak 2001). The second operationalization, termed *Political Expression Scale*, credits respondents for expressing a valid view. In this approach to scoring answers, definite answers—no matter if they are correct or incorrect—are scored 1, and DKs are given the score 0.^4^ Last, the *Political Accuracy Scale* compiles the proportion of the expressed opinions that are correct to address the concern that women’s and men’s propensity to guess is different. For example, a respondent getting one answer correct, one incorrect, and answering DK to one item scores 50 percent. All three scales are averaged so that responses range from 0 to 1.

### Individual-Level Factors

The standard predictors of political knowledge are generally based on individual attributes and resources. They refer to age, education, income, media attentiveness, political interest, socialization, and other equivalent indicators underlying political motivation such as mobilization in social groups (e.g., religious groups and political parties; Banwart 2007; Baxter and Lansing 1983; Bennett and Bennett 1989; Flora and Lynn 1974; Gidengil and Thomas 2008; Jennings 1983; Price and Zaller 1993; Wolak and McDevitt 2011). The following analyses will therefore include gender, age, education, income, and whether the respondent has voted (as a proxy for political interest, as no variable tapping into political interest was available in all three modules of the CSES). To test for the effects of group membership, the following indicators will be included: two items measuring the concept of political efficacy—“the feeling that individual political action does have, or can have, an impact on the political process, i.e., that it is worthwhile to perform one’s civic duties” (Campbell, Gurin, and Miller 1954, 187)—and whether respondents consider themselves close to a political party.^5^

### Macro-Level Factors

#### Question structure and content

As the CSES permitted national collaborators to ask questions about political knowledge according to their own standards in Modules 1 through 3, pooling the studies permits drawing from close to three hundred different items measuring political knowledge.^6^ This large variance makes it possible to analyze the effects of survey instruments on political knowledge.^7^ A handful of items regarding female politicians were included in certain countries. Although the scarcity of these items will make analyses including such questions more problematic, the following will incorporate a variable that indicates when indices contain at least one question pertaining to a female politician.

Moreover, items differ in their focus. The majority of questions target domestic politics in each of the countries, but some questions refer to the European Union and other international organizations.^8^ The analyses will thus contain an indicator identifying whether question batteries focus on domestic or international politics. Last, the question format varies from study to study: we find multiple-choice items, open-ended questions, and questions in the “true/false” format. These variations will allow controlling for question format across batteries of items, as the role of DK answers can be different in these situations (Luskin and Bullock 2011; Sturgis, Allum, and Smith 2008).

#### Societal factors

Although only few contributions integrating contextual predictors exist, recent research shows that the importance of individual-level motivational factors varies across media contexts (Iyengar et al. 2010). Some of the macro-level factors that underpin the level of women’s political representation, such as the presence of women in the workforce (Iversen and Rosenbluth 2008; Rosenbluth and Salmond 2006; Siaroff 2000), timing of enfranchisement, the importance of religion and types of religious affiliations (Inglehart and Norris 2003; Welch and Studlar 1986), and stereotypes and widely shared ideas about gender roles (Dahlerup 2006), could also be influential in explaining differing patterns of interest in politics. To encapsulate the degree to which countries adhere to traditional gender roles, the ensuing analyses will include the level of representation of women in each legislature included in the study (Inter-parliamentary Union [IPU] 2014). If the presence of women in parliaments can be hypothesized to influence the level of political engagement (Karp and Banducci 2008), which is one of the main determinants of political knowledge, we should expect differences in levels of political knowledge across countries, depending on how active and well represented women are in politics.

#### Institutional factors

Kittilson and Schwindt-Bayer (2012) argue that electoral institutions and their outcomes have a mediating effect on gender gaps in political engagement. In an analysis of twenty-eight countries, they find that levels of women’s legislative representation and the degree of proportionality of electoral rules are key factors explaining gender gaps in political knowledge. To address the potential influence of electoral rules on incentives for parties to mobilize women and, in turn, to boost levels of political knowledge, the following includes a measure of Gallagher’s (1991) least square index, the average district magnitude (log), and effective number of electoral parties for each election considered.^9^

#### Additional controls

A set of previous research has demonstrated that differences in cross-national contexts, for example, the level of income inequality, explain differences in overall levels of political knowledge between countries (Gordon and Segura 1997; Grönlund and Milner 2006). Although there are few reasons to hypothesize that such factors would affect men and women differently, the following analyses will comprise a control for the overall level of political knowledge in each country. Including the aggregate level of correct responses for each country helps to account for the possibility that the overall level of knowledge, or level of difficulty, influences men and women differently. Last, this study controls for survey mode to verify whether answers provided in differing interview environments, that is, face-to-face, telephone, and self-administered questionnaires, are affected by this structural component (Ansolabehere and Schaffner 2014).

## Analyses

### Macro-Level Analyses

**Figure 1.**
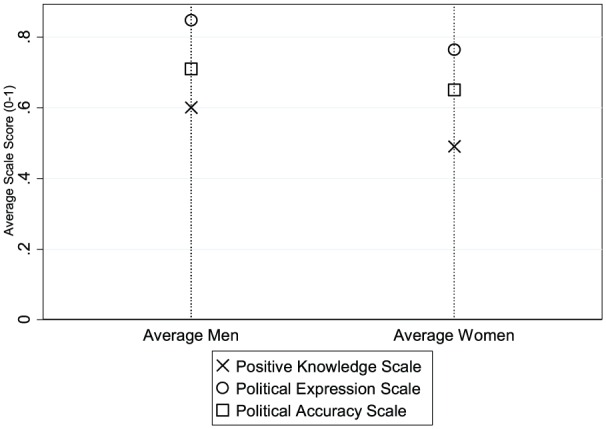
Distribution of men and women on three factual political knowledge scales using CSES Modules 1–3. Graph presents difference in mean score on political information between men and women in each 107 election study (CSES). CSES = Comparative Study of Electoral Systems.

The distribution of men’s and women’s scores on the three factual political knowledge scoring schemes, as illustrated in Figure 1, confirms the existence of an aggregate gender gap in factual political knowledge, no matter how the answers were coded. Women exhibit poorer scores, on average, in both the Political Expression Scale and Political Accuracy Scale. Women therefore tend to take recourse to the DK category more often than men and are also less accurate when they provide a definite answer. Combining these two factors—lower propensity to guess and lower accuracy rates—leads to sizable differences in the Positive Knowledge Scale, as highlighted in Figure 1. The differences between men and women are largest in the operationalization of the concept that code DK answers as incorrect (mean difference = 0.11, *SE* = 0.002) and smallest when scored in levels of accuracy (mean difference = 0.06, *SE* = 0.002).^10^ Women are therefore likely to be systematically penalized under coding schemes where DKs are scored as incorrect answers.

**Figure 2.**
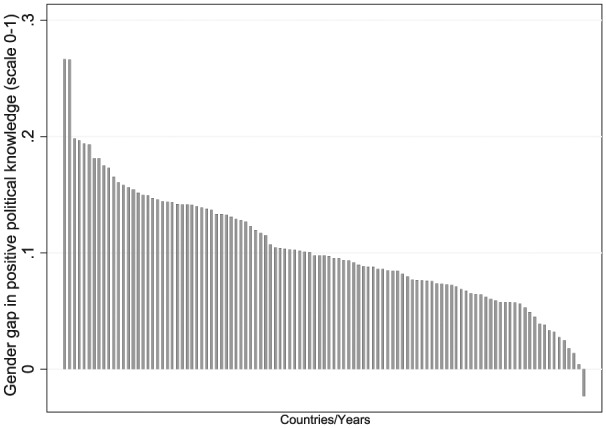
Distribution of the size of the gender gap on the Positive Knowledge Scale in 107 post-election surveys between 1996 and 2011. Graph presents difference in mean score on political information between men and women in each 107 election study (CSES)—Positive Knowledge Scale. CSES = Comparative Study of Electoral Systems.

Disaggregating these figures by countries, Figure 2 illustrates the distribution of the size of the gender gap in political information in 107 election studies between 1996 and 2011 (Positive Knowledge Scale). All election studies—with the exception of Chile in 2005^11^—display a gap in political knowledge between men and women in the favor of men. Yet the differences displayed in Figure 2 are not statistically significant in a handful of cases. For instance, Chile (2005), Japan (2004), Romania (1996), Australia (1996), and Sweden (2006) do not show significant differences between the political knowledge scores obtained by men and women (see the online appendix, Table II, for detailed difference of means tests). Perhaps most interesting is the large variation in the size of these gaps across countries. Given that scores can only range from 0 to 1, some of the gaps between men and women are very large for a particular group of countries. The largest difference between women and men is 0.27 in Greece (2009). Moreover, Switzerland (1999, 2003, and 2007) and Taiwan (1996, 2004, and 2008) consistently register some of the largest gaps, with, on average, 0.18 points difference between men and women. The fact that these cases exhibit steady scores provides evidence of substantive differences in levels of political information that are robust across different samples and survey questions.

Moreover, many countries appearing in more than one study achieve inconsistent scores over different elections: although the gender gap in Finland in 2011 is negligible (0.03), the registered gaps between men and women in political knowledge were 0.06 and 0.15 in 2007 and 2003, respectively, which indicates high fluctuations over different elections. One possible reason is that the battery of questions changed in content and format in each election study. A similar scenario materializes in Mexico, where scores range from 0.06 to 0.14 over the four elections contained in the three CSES modules despite using an almost identical battery of questions—in both content and format—over the years. Such fluctuations cast doubt that the standard instruments measuring political knowledge, contained in surveys, always produce reliable measurements of the target concept and serve as a reminder that caution should be exercised when interpreting results from single cases.

Although research on the determinants of gender gaps in political knowledge hitherto focused on individual-level factors, large variations between countries point to the potential presence of macro-level factors accounting for aggregate knowledge differentials between men and women. For instance, stereotypes and widely shared ideas about gender roles, which could have led to different forms of socialization between men and women, may possibly result in different levels of political involvement and political knowledge. Table 1 presents a series of ordinary least squares (OLS) regressions estimating the aggregate size of the gender gap in each country included in the study using the three different operationalizations of political knowledge described in the previous section.

**Table 1.** OLS Regression Models Estimating the Aggregate Size of the Gender Gap in Political Knowledge.

	Positive knowledge *b* (*SE*)	Expression scale *b* (*SE*)	Accuracy scale *b* (*SE*)
Percent women in parliament	0.000	−0.000	0.000
	(0.00)	(0.00)	(0.00)
Institutions			
Gallagher’s least square disproportionality index	0.002**	0.002	0.001
	(0.00)	(0.00)	(0.00)
Effective number of parties	0.001	0.002	−0.001
	(0.00)	(0.00)	(0.00)
District magnitude (log)	0.003	0.002	−0.000
	(0.00)	(0.00)	(0.01)
Question format			
True/false (ref.)	—	—	—
Multiple choice	−0.047*	0.023	−0.049*
	(0.03)	(0.04)	(0.02)
Open	0.014	0.007	0.022
	(0.02)	(0.02)	(0.02)
Mixture of formats	0.003	0.018	0.018
	(0.02)	(0.02)	(0.02)
Content of questions			
National focus (ref.)	—	—	—
National and international	0.028***	0.037***	0.000
	(0.01)	(0.01)	(0.01)
One question containing gender-specific knowledge	0.003	0.015	−0.037
	(0.02)	(0.02)	(0.02)
Mean of country score (question difficulty)	−0.036**	−0.031	−0.038***
	(0.01)	(0.03)	(0.01)
Survey mode			
Face to face (ref.)	—	—	—
Telephone	0.023	0.038**	0.009
	(0.02)	(0.02)	(0.02)
Self-administered	−0.003	−0.000	−0.001
	(0.02)	(0.02)	(0.02)
Mixture	0.020	0.012	0.007
	(0.02)	(0.02)	(0.02)
Constant	0.113***	0.101	0.099**
	(0.04)	(0.08)	(0.04)
Observations	102	81	102
*R* ^2^	.264	.307	.188

Models contain jackknifed standard errors in brackets. Political Expression Scale excludes countries where DK was not offered to respondents as a category. OLS = ordinary least squares; DK = don’t know.

* *p* < .10. **p < .05. ***p < .01.

The percentage of women in a country’s lower house does not have a statistically significant impact on the dependent variables: the environments in which women are less empowered are not necessarily those where the knowledge differences between men and women are the most pronounced, which contradicts the arguments in previous research concerning the effects of socioeconomic and cultural contexts.^12^ The institutional variables—Gallagher’s least square index of disproportionality, the effective number of political parties, as well as district magnitude—also fail to achieve a statistically significant impact across most model specifications, which is at odds with Kittilson and Schwindt-Bayer’s (2012) finding that more inclusive electoral contests lead to increased political engagement of women compared with men. Although more disproportionality is associated with larger gender gaps in positive knowledge, this parameter estimate loses its statistical significance when a single observation, Albania 2005, is pulled from the analyses.

Turning to survey instruments, we notice that multiple-choice questions, compared with “true/false” and open-ended questions, appear to reduce the gap between men and women in both the positive knowledge and accuracy scales. Yet, question format does not affect aggregate gender gaps in propensity to express a view (expressive knowledge). Batteries of questions containing items regarding international organizations, officeholders of other countries, and phenomena external to the national context seem to lead to larger differences between men and women, in comparison with batteries of items entirely focusing on domestic issues. Last, perhaps due to the small number of questions about female politicians, batteries containing at least one item measuring more gender-specific knowledge do not exercise significant influence on overall differences in scores between men and women. The item measuring question difficulty reveals that overall performance on item batteries affects the size of the gender gap in positive knowledge and accuracy but does not affect the gap in expressive answers.

Overall, the type of questions used, their content, and the level of difficulty, rather than social and institutional contexts, account for a large proportion of the variance in political knowledge gender gaps when counting correct answers and levels of accuracy. By contrast, the most important proportion of the disparity between men and women in expressive answers is attributable to survey mode and question content, rather than format and level of difficulty. The aggregate-level analyses therefore suggest that the different propensities of men and women to give both correct and accurate sets of answers hinge on similar factors, yet that a different group of factors underpins their propensity to express a valid opinion as opposed to DK.

### Multilevel Analyses

In the following section, I estimate three different multilevel ordered logistic models on the three alternative political knowledge scales to address the hierarchical structure of the data.^13^ Table 2 summarizes the covariates explaining the number of correct and expressive responses as well as their level of accuracy, including a series of interaction effects with gender.

**Table 2.** Multilevel Mixed-Effects Ordered Logistic Regressions Estimating Additive Political Knowledge Scores in Individuals.

	Positive knowledge^a^	Expression scale^a^	Accuracy scale^a^
	*b* (*SE*)	*b* (*SE*)	*b* (*SE*)
Micro-level covariates			
Gender (1 = *women*)	−0.385*	−0.424	−0.061
	(0.00)	(0.00)	(0.27)
Age	0.014***	0.244***	0.009***
	(0.00)	(0.02)	(0.00)
Education	0.274***	0.137***	0.181***
	(0.02)	(0.02)	(0.02)
Income (quintiles)	0.158***	0.051***	0.094***
	(0.01)	(0.01)	(0.01)
Who is in power makes a difference	0.053***	0.056***	0.035***
	(0.01)	(0.01)	(0.01)
Who people vote for makes a difference	0.051***	0.082***	0.029***
	(0.01)	(0.01)	(0.01)
Closeness to a party (1 = *yes*)	0.069***	0.114***	0.029***
	(0.01)	(0.01)	(0.01)
Vote in current elections (1 = *yes*)	0.144***	0.012***	0.092***
	(0.01)	(0.00)	(0.01)
Macro-level covariates			
% of women in parliament	−0.012**	0.003	0.045*
	(0.00)	(0.03)	(0.02)
Survey mode			
Face to face (ref.)	—	—	—
Telephone	−0.008	−0.798	−0.073
	(0.08)	(0.57)	(0.10)
Self-administered	−0.094	−0.494***	0.027
	(0.08)	(0.10)	(0.03)
Mixture	−0.451***	−1.324*	−1.029***
	(0.09)	(0.72)	(0.13)
Questions			
Format: true/false (ref.)	—	—	—
Format: multiple choice	−0.310*	0.529	0.509
	(0.18)	(1.43)	(0.67)
Format: open	0.176*	−0.192	1.466***
	(0.10)	(0.80)	(0.41)
Format: mix	0.062	1.277	1.501***
	(0.08)	(1.44)	(0.08)
One gender-specific item (1 = *yes*)	−0.030	−0.610	1.420***
	(0.08)	(0.45)	(0.27)
Mean country score (difficulty)	2.182***	1.549***	2.645***
	(0.11)	(0.45)	(0.16)
Content: national focus (ref.)	—	—	—
Content: national and international	0.156***	−1.285**	−0.552***
	(0.06)	(0.59)	(0.12)
Institutions			
Gallagher’s disproportionality index	−0.010	0.034	0.042
	(0.01)	(0.04)	(0.05)
Effective number of parties (votes)	0.064*	−0.244	0.480***
	(0.03)	(0.20)	(0.05)
District magnitude (logged)	−0.144**	0.074	−0.912***
	(0.06)	(0.17)	(0.22)
Interactions with gender			
Female × % of women in parliament	−0.004	−0.002	−0.006*
	(0.00)	(0.00)	(0.00)
Female × One gender-specific item (1 = *yes*)	−0.037	−0.038	0.209
	(0.15)	(0.12)	(0.19)
Female × Mean country score on battery	0.062	−0.116	0.140*
	(0.08)	(0.16)	(0.08)
Female × Content: national and international	−0.168***	−0.183**	−0.052
	(0.06)	(0.09)	(0.08)
Female × Format: true/false (ref.)	—		—
Female × Format: multiple choice	0.465***		0.356***
	(0.16)		(0.12)
Female × Format: open	−0.068		−0.198*
	(0.09)		(0.10)
Female × Format: mix	−0.002		−0.167
	(0.10)		(0.12)
Female × Gallagher’s disproportionality index	−0.010	−0.011	−0.021**
	(0.01)	(0.01)	(0.01)
Female × Effective number of parties (votes)	−0.020	−0.031	−0.024
	(0.02)	(0.03)	(0.02)
Female × District magnitude (logged)	0.007	0.009	0.016
	(0.03)	(0.04)	(0.03)
Female × Face to face (ref.)	—	0.000	—
Female × Telephone	—	−0.246**	—
	—	(0.11)	—
Female × Self-administered	—	0.047	—
	—	(0.15)	—
Female × Mixture	—	−0.007	—
		(0.16)	
Cut/constant (omitted)			
Random component (country/election level)	0.101***	6.002***	2.251***
	(0.03)	(2.15)	(0.75)
Observations	100,188	75,766	95,730
Election studies	77	64	77
*R* ^2^	.11	.07	.07

Pseudo *R*^2^ estimated via standard ordered logistic regression (separate). Political Expression Scale excludes countries where DK was not offered to respondents as a category. DK = don’t know.

a Albania 2005 omitted.

* *p* < .10. **p < .05. ***p < .01.

The results from all models highlight the significant differences in the levels of political knowledge between men and women observed in the previous sections of this article. The micro-level covariates demonstrate significant resource differences in political knowledge across the citizens contained in CSES surveys, which is consistent with findings in the literature on the topic (Banwart 2007; Bennett and Bennett 1989; Delli Carpini and Keeter 1996; Dow 2009; Flora and Lynn 1974; Frazer and Macdonald 2003; Gidengil and Thomas 2008; Jennings 1983; Price and Zaller 1993; Wolak and McDevitt 2011). The results demonstrate that the likelihood of individuals reaching higher numbers of correct responses increases with age, education, and income. Higher levels of political efficacy (e.g., having voted, and/or identifying with a political party, that is, all indicators of political group involvement) also affect the likelihood of achieving higher numbers of correct responses, no matter how political knowledge is scored.

Turning to the interaction effects between gender and macro-level covariates, the models yield similar patterns as those uncovered in Table 1, hence lending additional confidence in the overall model specifications. The coefficient measuring the effect of women’s representation in each country’s lower house does not reach conventional levels of statistical significance in any model, meaning that this covariate does not affect political knowledge scores among men and women differently. In other words, if opportunity structures improve for women, political knowledge scores for women never increase enough to close the gap with men’s scores. This suggests that the effect of opportunity structures manifests itself in ways not captured by standard political knowledge questions. A similar development unfolds in the case of institutions where disproportionality achieves statistical significance on positive knowledge and accuracy scores when interacted with gender, albeit only on lower values of the dependent variable. How to explain the absence of a clear relationship between political institutions and the gender gap size? One possible reason is that a country-level measure of disproportionality, that is, the Gallagher index, cannot cope with the complexity of the specific mobilization incentives faced by parties of different size, ideological orientation, and competitiveness. In other words, the effect of electoral rules is probably too causally distant from individuals to hypothesize that they might have a distinct effect on men and women, and relies on a set of intervening mechanisms that cannot be measured at the country level.

Question content yields more insights into gendered differences in political knowledge, although not all elements hypothesized to play a role are influential. Batteries of questions containing at least one gender-specific item do not boost the scores of women on any outcome variable. However, given the almost trivial amount of these items in the body of questions fielded in the CSES, this result should not be considered a definitive answer. The substantive focus of questions also affects women and men differently: batteries of questions that contain items on international affairs negatively influenced the scores women obtained but had positive impacts on the sub-sample of men, thus serving to increase the gulf between genders in political knowledge. On this type of question, the role of DK is clear: content does not affect the accuracy of women’s answers but affects their likelihood of answering DK and thus also their positive knowledge scores negatively. Last, battery difficulty seemingly has no effect on women’s levels of positive knowledge, but once DKs are purged, we notice the normal pattern observed for men, namely that women are less accurate as batteries become more difficult.

Table 2 largely replicates the macro-level findings concerning question format from Table 1. Multiple-choice question formats affect women’s scores more positively than “true/false” formats on both the Positive Knowledge Scale and the Political Accuracy Scale, meaning that the penalty for answering DK built in the Positive Knowledge Scale does not wash out this effect. By contrast, there is no significantly different effect for men between “true/false” and multiple-choice questions. Also, women are less likely to give accurate series of answers than men on open-ended questions when compared with “true/false” formats. The question format therefore carries a sizable gendered impact on the scores of respondents. This is most visible when contrasting the positive knowledge scores, where DK is considered an incorrect response, with the level of accuracy of answers. These results substantiate existing research on the different psychological tendencies of men and women to guess (Atkeson and Rapoport 2003; Frazer and Macdonald 2003; Kenski and Jamieson 2000; Lizotte and Sidman 2009; Mondak and Anderson 2004; Mondak and Canache 2004), and justify the use of alternative specifications of political knowledge. Even when the potential for easy “lucky guessing” is diminished, such as in open-ended questions, men are still more accurate than women in correctly answering political knowledge questions.

Finally, as already outlined in the macro-level analyses, the covariates explaining scores on the Political Expression Scale differ from the two other operationalizations. The likelihood of providing an answer, correct or incorrect, as opposed to answering DK, hinges more on survey mode than on question format. Here, women are less likely to provide expressive answers via telephone than face to face with a survey interviewer, hinting that women were more at ease to answer DK in circumstances where they are not confronted directly with another person.

To sum up, patterns underpinning correct, accurate, and expressive answers to political knowledge questions vary in large measure. The substantial repercussions that the choices of survey content and mode have on final scores and potential explanations underline that more attention has to be paid to these differing structures of answering behavior.

## Discussion and Conclusion

Cognitive engagement is crucial for democratic citizenship. Yet it builds on a resource that is unequally distributed: political knowledge. This article contributes to the ongoing debate about causes and explanations of the gender gap. To this end, it systematically tests competing explanations using the broadest country coverage to date. Tracing causal relations on both micro- and macro-level allows for a more nuanced understanding of the mechanisms at work. In sum, this article sought to make two major contributions.

First, the results reveal that significant gender gaps in political knowledge exist in almost all countries under scrutiny irrespective of how scores are allocated to answers (positive, expressive, accurate). However, the gap is largest if DK answers are considered as incorrect answers. As women are more likely to choose this category than men, these scoring schemes, commonly used in research, systematically penalize women. Although the analyses presented here suggest that the different propensities of men and women to give both correct and accurate answers hinges on similar factors, the different scoring schemes used to code answers revealed that a different set of factors underpins their propensity to express a valid opinion as opposed to DK. These findings underline the importance of selecting scoring systems very carefully: without paying attention to the “response bias” characteristics of the DK category, valid comparisons between men and women can hardly be drawn.

Second, both the macro- and multilevel analyses conducted in this article provide persuasive evidence that individual-level as well as macro-level factors are shaping the size of the gender gap in political knowledge. Factors associated with larger gender gaps in the aggregate analyses also tend to affect the individual scores of men and women differently in the multilevel analyses. In short, this analysis of political knowledge gaps revealed that women and men react in different ways to framework conditions and survey content: women, for instance, are more likely to score high, when confronted with multiple-choice questions rather than with “true/false” or open-ended-format questions. By contrast and at odds with recent research, institutional and broader contextual variables failed to explain differences in levels of political knowledge between men and women in both the aggregate and multilevel analyses.

Two caveats have to be kept in mind: first, despite confirming the results of previous studies on the relevance of individual-level factors, adding macro-level variables was not sufficient to remove the remaining differences between men and women in political knowledge scores: a large part of the variance remains unexplained. This raises important implications for further testing in seeking to explain the remaining gap. Second, the analyses reveal large differences in gender gap size within individual countries over time. Such fluctuations cast doubt on the validity of the standard instruments measuring factual political knowledge. Factual knowledge items contain an element of randomness, due to lucky guessing, that is large enough to create substantial variations over time, even in ideal testing conditions where identical survey instruments are used. This random component may account for the puzzling finding that differences in opportunity structures for women—irrespective of the operationalization chosen in this article—do not seem to make an impact on either aggregate gender gaps or individual scores.

The findings presented in this contribution add a new dimension to the issue of cross-national comparison of survey measurements. Nevertheless, the issue of comparability of survey items remains problematic between countries and over time: are questions too different to allow for meaningful cross-national comparisons (Elff 2009)? Are questions too unstable, considering their dependence on unique contexts, to enable comparison over time? What would be the implication of fielding two batteries of questions, one for women and one for men, as proposed by Dolan (2011)? Although this article did not intend to model absolute knowledge levels but rather differences between groups inside single countries at single points in time, the issue of measurement equivalence is arguably less problematic. Yet eliminating some of these differences with uniform batteries of questions could serve to make the results of these inquiries more reliable cross-nationally and cross-temporally. All the same, the questions raised above still lack answers and thus pose a challenge to further research.

## Supplementary Material

Supplementary material

## Notes

1. Kittilson and Schwindt-Bayer (2012) investigated the effects of electoral rules on gender gaps in political knowledge. A recent study by Marta Fraile (2014) has looked at differences in political knowledge in European countries; however, the study does not look at macro-level covariates that could influence gender differences in political knowledge.

2. The person asking the questions can also have an impact (see McGlone, Aronson, and Kobrynowicz 2006).

3. It is worthwhile underscoring that as there are only three political knowledge items fielded per study, it is not possible to perform rigorous individual item analyses to choose the questions that allow making sharper discrimination between respondents, as suggested by Delli Carpini and Keeter (1993).

4. For this operationalization, election studies in which the option “don’t know” (DK) was not offered to respondents, or already coded “missing” by country collaborators, were excluded from the analyses. This was mostly the case in Comparative Study of Electoral Systems (CSES) Module 1 countries.

5. An indicator of level of religiosity was considered but ultimately dropped due to data coverage issues, as many national election study teams are not permitted to ask respondents about their religious beliefs and practices.

6. It is also important to note that most countries with repeated studies across modules have fielded different questions over time, which makes pooling all the more advantageous.

7. Besides, it remains doubtful whether full standardization of political knowledge items will make them more comparable in practice, for example, if the requirements for configural, metric, or scalar invariance would be reasonably met, as the difficulty of certain factual items is dependent upon context (Turgeon 2008).

8. Although we know that practical questions about benefits and services that are directly relevant to citizens’ daily lives are types of questions where women tend to perform better, this kind of item was not asked in the studies that compose the CSES.

9. Gallagher’s least squares index measures the disparity between vote shares and seat shares according to the following formula: ${\mathrm{LS}}_{q}=\sqrt{\frac{1}{2}}\sum_{i=1}^{n}{\left(V_{i}{-S}_{i}\right)}^{2}$. The measure of district magnitude was drawn from Beck et al. (2001). The effective number of electoral parties was calculated via the formula provided by Gallagher and Mitchell (2008).

10. Calculated from standardized scores to allow direct comparison.

11. The difference of means between men and women is not statistically significant at the 10 percent level in this group of observations.

12. Models (not shown) using alternative measures such as the gender inequality index (GII; United Nations Development Programme [UNDP] 2013), which measures gender disparities in reproductive health, political empowerment, and labor-market participation, and the World Economic Forum’s (WEF; 2013) Global Gender Gap Index (GGGI), tracing the magnitude of gender-based disparities in economic participation and opportunity, educational attainment, health issues, and political empowerment, did not yield different results.

13. Likelihood tests (not shown) indicate that there is enough variability between election studies to favor mixed-effects ordered logistic regressions over standard ordered logistic regressions.
