# Supplementary material for: Cross-National Gender Gaps in Political Knowledge: How Much Is Due to Context?
Source: Polit Res Q. 2016 Apr 13;69(3):391–402. doi: 10.1177/1065912916642867 (PMC4968032; doi:10.1177/1065912916642867)
Supplement: Supplementary material [file Supplemental_Material.pdf]

**Appendix**

TABLE I. Countries/years included

| Country | Years | | | | Country | Years | | | | |
|---|---|---|---|---|---|---|---|---|---|---|
| Albania | 2005 | | | | Korea | 2004 | | | | |
| Australia | 1996 | 2004 | 2007 | | Kyrgyzstan | 2005 | | | | |
| Austria | 2008 | | | | Latvia | 2010 | | | | |
| Belgium | 2003 | | | | Mexico | 1997 | 2000 | 2003 | 2006 | 2009 |
| Belgium (Flanders) | 1999 | | | | Netherlands | 1998 | 2002 | 2006 | 2010 | |
| Brazil | 2002 | 2006 | 2010 | | New Zealand | 1996 | 2002 | 2008 | | |
| Canada | 1997 | 2004 | 2008 | | Norway | 1997 | 2001 | 2005 | 2009 | |
| Chile | 2005 | 2009 | | | Peru | 2006 | 2011 | | | |
| Croatia | 2007 | | | | Philippines | 2004 | 2010 | | | |
| Czech Republic | 1996 | 2002 | 2006 | 2010 | Poland | 1997 | 2001 | 2005 | 2007 | |
| Denmark | 2007 | | | | Portugal | 2002 | 2005 | 2009 | | |
| Estonia | 2011 | | | | Romania | 1996 | 2004 | 2009 | | |
| Finland | 2003 | 2007 | 2011 | | Russia | 2004 | | | | |
| France | 2002 | 2007 | | | Slovakia | 2010 | | | | |
| Germany | 1998 | 2002 | 2005 | 2009 | Slovenia | 2004 | | | | |
| Great Britain | 1997 | 2005 | | | South Africa | 2009 | | | | |
| Greece | 2009 | | | | South Korea | 2008 | | | | |
| Hong Kong | 1998 | 2004 | 2008 | | Spain | 1996 | 2000 | 2004 | 2008 | |
| Hungary | 1998 | 2002 | | | Sweden | 1998 | 2002 | 2006 | | |
| Iceland | 2007 | 2009 | | | Switzerland | 1999 | 2003 | 2007 | | |
| Ireland | 2002 | 2007 | | | Taiwan | 1996 | 2001 | 2004 | 2008 | |
| Israel | 1996 | 2003 | 2006 | | Thailand | 2007 | | | | |
| Italy | 2006 | | | | Ukraine | 1998 | | | | |
| Japan | 1996 | 2004 | 2007 | | USA | 1996 | 2004 | 2008 | | |

47 countries

TABLE II. Size of gender gap in each study on the positive knowledge scale, with levels of significance of difference of means test between men and women.

| *Country year* | *Gender gap* | *Country year* | *Gender gap* | *Country year* | *Gender gap* |
|---|---|---|---|---|---|
| CHL_2005 | -.0231 | CAN_2008 | .0769*** | ESP_2004 | .1311*** |
| JPN_2004 | .0041 | HGK_2008 | .0795*** | SVN_2004 | .1332*** |
| ROU_1996 | .0137 | NZL_2002 | .0845*** | HUN_1998 | .1333*** |
| AUS_1996 | .0179 | IRL_2002 | .0846*** | TWN_2001 | .1327*** |
| AUS_2007 | .0248* | UKR_1998 | .0847*** | MEX_2000 | .1371*** |
| FIN_2011 | .0276* | NOR_2009 | .0885*** | HRV_2007 | .1381*** |
| SWE_2006 | .0319 | JPN_2007 | .0861*** | NLD_2010 | .1401*** |
| BELW1999 | .0334 | PRT_2009 | .0862*** | POL_2005 | .1414*** |
| CZE_1996 | .0382** | ITA_2006 | .0881*** | PER_2011 | .1411*** |
| PHL_2010 | .0387** | USA_1996 | .0881*** | POL_1997 | .1415*** |
| AUT_2008 | .0452** | NOR_1997 | .0896*** | PRT_2002 | .1436*** |
| ROU_2004 | .0575*** | BEL_2003 | .0917*** | FRA_2002 | .1439*** |
| CZE_2002 | .0491*** | CAN_1997 | .0936*** | DEU_1998 | .1440*** |
| CZE_2010 | .0522*** | CZE_2006 | .0932*** | MEX_2003 | .1389*** |
| KOR_2008 | .0686*** | BRA_2010 | .0952*** | FIN_2003 | .1459*** |
| NZL_2008 | .0561*** | USA_2008 | .0952*** | PRT_2005 | .1420*** |
| IRL_2007 | .0576*** | NOR_2001 | .1009*** | CAN_2004 | .1473*** |
| KGZ_2005 | .0574*** | HKG_2000 | .0972*** | GBR_2005 | .1496*** |
| FIN_2007 | .0591*** | NZL_1996 | .0975*** | TWN_2004 | .1493*** |
| ZAF_2009 | .0601*** | AUS_2004 | .0977*** | DEU_2002 | .1517*** |
| LVA_2010 | .0577*** | ESP_2000 | .0977*** | SWE_2002 | .1544*** |
| ISR_1996 | .0621* | POL_2007 | .1018*** | POL_2001 | .1563*** |

| | | | | | |
|---|---|---|---|---|---|
| MEX_2006 | .0640*** | BRA_2002 | .1036*** | FRA_2007 | .1582*** |
| RUS_2004 | .0644*** | NLD_2006 | .1028*** | DNK_2007 | .1607*** |
| MEX_2009 | .0710*** | NOR_2005 | .1027*** | GBR_1997 | .1655*** |
| HKG_2004 | .0674*** | BRA_2006 | .1043*** | TWN_2008 | .1731*** |
| KOR_2004 | .0735*** | ROM_2009 | .1044*** | BELF1999 | .1751*** |
| EST_2011 | .0733*** | SWE_1998 | .1071*** | CHE_2003 | .1812*** |
| HUN_2002 | .0727*** | DEU_2009 | .1006*** | ESP_1996 | .1814*** |
| NLD_2002 | .0724*** | USA_2004 | .1151*** | PER_2006 | .1942*** |
| ISL_2009 | .0817*** | ISL_2007 | .1171*** | CHE_2007 | .1932*** |
| THA_2007 | .0650*** | MEX_1997 | .1230*** | ALB_2005 | .1966*** |
| DEU_2005 | .0759*** | SVK_2010 | .1195*** | CHE_1999 | .1984*** |
| ESP_2008 | .0763*** | HKG_1998 | .1269*** | TWN_1996 | .2663*** |
| ISR_2006 | .0755*** | NLD_1998 | .1290*** | GRC_2009 | .2666*** |
| PHL_2004 | .0767*** | ISR_2003 | .1283*** | | |

NOTE: Table presents difference in mean score on political information (positive knowledge 0-1) between men and women in each election study.

$^{*}$ $p < 0.05$, $^{**}$ $p < 0.02$, $^{***}$ $p < 0.01$.
